# Anemia in patients with diabetic foot ulcer and its impact on disease outcome among Nigerians: Results from the MEDFUN study

**DOI:** 10.1371/journal.pone.0226226

**Published:** 2019-12-17

**Authors:** Ibrahim D. Gezawa, Ejiofor T. Ugwu, Ignatius Ezeani, Olufunmilayo Adeleye, Innocent Okpe, Marcelina Enamino

**Affiliations:** 1 Division of Endocrinology, Diabetes and Metabolism, Department of Medicine, Bayero University, Kano, Nigeria; 2 Division of Endocrinology, Diabetes and Metabolism, Department of Medicine, Enugu State University of Science and Technology, Enugu, Nigeria; 3 Division of Endocrinology, Diabetes and Metabolism, Department of Medicine, Federal Medical Center, Umuahia, Nigeria; 4 Division of Endocrinology, Diabetes and Metabolism, Department of Medicine, Lagos State University, Lagos, Nigeria; 5 Division of Endocrinology, Diabetes and Metabolism, Department of Medicine, Ahmadu Bello University, Zaria, Nigeria; 6 Division of Endocrinology, Diabetes and Metabolism, Department of Medicine, Federal Medical Center, Keffi, Nigeria; University Magna Graecia of Catanzaro, ITALY

## Abstract

**Background:**

Diabetes is a life-long and debilitating disease that is fraught with both acute and chronic complications. Of particular concern to sufferers of the disease is the development of foot problems. These problems range from foot deformities to slowly healing or non-healing ulcers (that may necessitate amputation) and in the worst-case scenario, to death. Identification and prompt treatment of comorbid conditions, such as anemia may improve outcome in patients with diabetic foot ulcers (DFU). We determined the prevalence of anemia in Nigerians with DFU and its impact on disease outcome.

**Methods:**

We prospectively followed 336 patients with diabetes hospitalized for DFU and managed by a multidisciplinary team until discharge or death. Demographic and diabetes-related information and ulcer characteristics were documented. We evaluated each patient for neuropathy, vasculopathy and medical co-morbidities. Relevant laboratory and imaging studies were performed. We present the results of the sub-group analysis of patients with anemia to determine its prevalence and impact on disease outcome in patients with DFU in the MEDFUN study.

**Results:**

Anemia was detected in 180(53.6%) subjects with 88(48.9%) of them requiring blood transfusion. Significant demographic and clinical determinants of anemia were ulcer duration more than one month prior to hospitalization (p<0.009), PAD (p<0.001) and presence of gangrene (p<0.001). The comorbid conditions that were significantly associated with anemia included proteinuria (p<0.003), osteomyelitis (p<0.006), moderate (p<0.002) as well as severe (p<0.001) vascular stenosis, history of stroke (p<0.014) and renal impairment (p<0.002). Anemia was significantly associated with poor wound healing (p<0.009), amputation (p<0.036) and risk of death (p<0.034).

**Conclusion:**

We detected anemia in more than half of our cohort with DFU. We found significant association between anemia and poor wound healing, amputation and mortality among our studied subjects. Future studies should explore whether prompt correction of anemia in subjects hospitalized for DFU would improve outcome.

## Introduction

Despite global efforts to tame the tide of rising epidemic of non-communicable diseases, the prevalence of diabetes has continued to soar. Available data suggest that the prevalence of diabetes would rise from 425 million in 2017 to 625 million by the year 2045[1]. With a projected 156% increase in the prevalence of diabetes, Sub-Saharan Africa (SSA) is expected to be worst hit by the looming epidemic.

Diabetes mellitus (DM) is a major cause of morbidity and mortality worldwide [2]. Persistent hyperglycemia, the hallmark of uncontrolled DM, causes generalized vascular damage targeting the heart, eyes, kidneys and nerves making DM one of the leading causes of cardiovascular disease, blindness, kidney failure and lower limb amputation in the world [1]. The mortality associated with DM is equally high. According to the International Diabetes Federation (IDF), approximately 4.0 million people aged between 20 and 79 years died from DM in 2017, equivalent to one death every eight seconds [1].

Diabetic foot ulceration (DFU) is a serious and life threatening complication of DM. According to estimates, up to 25% of people living with diabetes (PLWD) are at risk of developing DFU during their lifetime [3]. The prevalence of DFU ranges from 4.6% in Jordan [4] to 15% in the United States [5]. Nigeria, with the greatest burden of DM in SSA, is reported to have a prevalence of DFU of about 10%, with a quarter of newly diagnosed patients presenting with the disease [6]. Several factors contribute to the development of DFU. Apart from vasculopathy (micro- and macrovascular disease), poor glycemic control, foot deformity, altered foot biomechanics; polyneuropathy, active infection, inflammation and impaired immunity also play major roles in the pathophysiology of DFU [7]. These factors may also influence outcome in affected patients.

Studies have shown that anemia is twice as common in patients with DM compared with those without the disease [8, 9]. In those with DM, anemia predisposes to the development of stroke, ischemic heart disease, hypertension and nephropathy [10]. Anemia in patients with DFU is associated with adverse outcomes [11]. There is paucity of studies on anemia in patients with DFU in Nigeria. We therefore set out to determine the prevalence of anemia and its impact on outcome in Nigerians with DFU.

## Materials and methods

### Study design and setting

The Multi-center Evaluation of Diabetic Foot Ulcer in Nigeria (**MEDFUN**) was an observational study conducted between March 2016 and April 2017 in six tertiary healthcare institutions across Nigeria.The centres included Lagos State University Teaching Hospital (Southwest Nigeria), Enugu State University Teaching Hospital (Southeast Nigeria), Aminu Kano Teaching Hospital (Northwest Nigeria); Ahmadu Bello University Teaching Hospital Zaria (Northwest Nigeria), Federal Medical Centre Keffi (North Central Nigeria) and Federal Medical Centre Umuahia (Southeast Nigeria). These tertiary hospitals serve as referral centers for primary and secondary healthcare facilities within and outside their geopolitical zones.

### Study subjects and sampling

Our study subjects consisted of consecutive patients with type 1 diabetes (TIDM) or type 2 diabetes (T2DM) hospitalized for DFU at any of the participating centers. Prior to recruitment, we explained the study to the patients including the aim of the study, procedure, benefits, risks and their right not to participate if they so wish. The participants were allowed sufficient time to ask questions. Subsequently, we obtained verbal consent from each of the patients many of whom were not literate. We ensured confidentiality at all stages by recording patients’ information using a unique coding system that incorporates patient’s initials and an assigned number.

In this study, we considered patients who have been dependent on insulin for the control of their diabetes since diagnosis as having T1DM, while T2DM refers to those controlled on oral antidiabetic drugs with or without insulin since diagnosis. We excluded patients with other forms of diabetes, pregnant women and those with hemoglobinopathies.

### Study procedure

We prospectively followed 336 patients with diabetes (both type 1 and 2) hospitalized for DFU and managed by a multidisciplinary team until discharge or death. Demographic and diabetes-related information and ulcer characteristics were documented. We evaluated each patient for peripheral neuropathy, diagnosed based on loss of pressure perception to 10g Semmes-Weinstein monofilament test or diminished vibration sense using 128Hz tuning fork, while peripheral artery disease (PAD) was diagnosed based on impalpable dorsalis pedis and/or posterior tibial artery pulsations on manual palpation or significant arterial narrowing (>50%) on Doppler ultrasonography of the lower limbs. We also assessed each patient for medical co-morbidities such as hypertension, hyperglycemic emergencies, stroke, hypoglycemia, kidney disease, cardiac failure and anemia.

Relevant laboratory investigations [full blood count, renal function test and glycated hemoglobin (HbA1c)] and imaging studies were performed. Anemia was defined as an Hb<12g/dL. Details of the MEDFUN study protocol has been published elsewhere [12]. We present the results of the sub-group analysis of patients with anemia to determine its prevalence and impact on disease outcome in patients with DFU in the MEDFUN study.

### Ethical approval

The research and ethics committees of Enugu State University Teaching Hospital, Enugu State, South-Eastern Nigeria; Lagos State University Teaching Hospital, Lagos State, in the South-West, Aminu Kano Teaching Hospital, Kano State, in the North-West, Ahmadu Bello University Teaching Hospital, Zaria, Kaduna State, in the North-West, Federal Medical Center Keffi, in the North-Central and Federal Medical Center, Umuahia, in the South-East, approved the study protocol.

### Statistical analysis

We used the Statistical Package for Social Sciences (IBM version 23.0; SPSS Inc., Chicago, IL, USA) to analyze data collected. Numbers and percentages or means and standard deviations were computed for categorical and continuous variables, respectively. For the current sub-analysis we performed unadjusted associations between demographic, clinical and laboratory variables and anemia using the Chi-Square statistics for categorical variables and Student t-test for continuous variables.

Univariate logistic regression was performed for each variable of interest to identify the determinants of anemia by calculating their crude odds ratios (ORs) and 95% confidence intervals (CI). All the variables that significantly predicted anemia at this univariate level of analysis were subjected to a multivariate logistic regression. Statistical significance was assumed at P <0.05.

## Results

Of the 336 subjects recruited for the study, 185(55.1%) were males. The mean age of the subjects was 55.9±12.5 years. Majority [323(96.1%)] of the subjects had type 2 diabetes. The mean duration of DM among the subjects was 8.5 ± 5.7years. The mean duration of foot ulcer prior to admission was 39 days with a range of 28–54 days. Wound infection was present in 258 (76.8%) subjects. One-third of the subjects had Wagner grade 4 ulcers (Table 1). Anemia was detected in 180(53.6%) subjects, with 88(48.9%) requiring blood transfusion.

**Table 1:** Socio-demographic and clinical characteristics of the subjects.

Variable	Overall
Age (years)	55.9 ± 12.5
Gender (male)	185 (55.1%)
Diabetes type (type 2)	323 (96.1%)
Diabetes duration (years)	8.5 ± 5.7
Glycated hemoglobin (%) (n = 296)	9.6 ± 1.9
Receiving diabetes care at the study centers prior to admission	95 (28.3%)
Ever received foot care education since diagnosis of diabetes	87 (25.9%)
Cigarette smoking (current smokers)	17 (5.1)
Duration of ulcer before admission (days)	39 (28–54)
Previous history of ulcer	96 (28.6%)
Presence of wound infection	258 (76.8%)
Ulcer grade (Wagner)	
Grade 1	13 (3.9%)
Grade 2	57 (17.0%)
Grade 3	88 (26.2%)
Grade 4	124 (36.9%)
Grade 5	54 (16.1%)
Co-morbid complications	
Hypertension	191 (56.8%)
Shock	40 (11.9%)
Anemia	180 (53.6%)
Required blood transfusion (n = 180)	88 (48.9%)
Hyperglycemic emergency	123 (36.6%)
Hypoglycemia	33 (9.8%)
Cardiac failure	23 (6.8%)
Renal impairment	66 (19.6%)
Stroke	32 (9.5%)

Table 2 shows the demographic and clinical determinants of anemia among the study subjects. Ulcer duration of ≥1month (OR = 1.89: 95%CI = 1.177–3.04), presence of peripheral arterial disease (OR = 2.14: 95%CI = 1.385–3.316) and gangrene (OR = 2.36: 95%CI = 1.522–3.66) were the significant determinants of anemia identified. The comorbid conditions significantly associated with anemia (Table 3), included proteinuria (OR = 2.04: 95%CI = 1.285–3.24), osteomyelitis (OR = 2.01: 95%CI = 1.218–3.34), severe vascular stenosis (OR = 4.15: 95%CI = 1.803–9.56), moderate vascular stenosis (OR = 2.48: 95%CI = 1.39–4.42), stroke (OR = 2.84: 95%CI = 1.24–6.53) and renal impairment (OR = 2.57: 95%CI = 1.43–4.60). On multivariate logistic regression analysis, none of these factors emerged as an independent predictor of anemia (Table 5). The relationship between anemia and DFU treatment outcomes is shown in (Table 4). Significant association was found between anemia and wound healing. Patients with anemia were less likely to have a satisfactory wound healing (OR = 0.52: CI = 0.32–0.85) compared with those without anemia. Conversely, hospitalized DFU patients with anemia were more likely to die when compared with those without anemia (OR = 1.82: 95%CI = 1.05–3.17). Our result also shows that patients with anemia were more likely to have amputation (OR = 1.67: 95%CI = 1.04–2.69) compared with those without anemia. The outcome of a multivariate logistic regression to determine the independent predictors of anemia among all the candidate univariate predictors of anemia is shown in Table 5. None of the variables that predicted anemia at the bivariate level of analysis emerged as an independent predictor when subjected to multivariate regression, suggesting that the predictors of anemia do not operate independent of one another but rather in a multifactorial, but synergistic manner.

**Table 2:** Demographic and clinical determinants of anemia.

	Anemia		P value	OR	95% C.I for OR
	Yes n (%)	No n (%)			
***Age (years)***					
<45 (reference)	22 (45.8)	26 (54.2)			
45–64	104 (52.0)	96 (48.0)	0.443	1.280	0.681–2.409
≥65	54 (61.4)	34 (38.6)	0.083	1.877	0.921–3.824
***Gender***					
Male	99 (53.5)	86 (46.5)	0.981	0.995	0.646–1.531
Female	81 (53.6)	70 (46.4)			
***Type of diabetes***					
Type 1	4 (30.8)	9 (69.2)	0.105	0.371	0.112–1.230
Type 2	176 (54.5)	147 (45.5)			
***Duration of diabetes***					
<10 years (reference)	127 (50.8)	123 (49.2)			
11–20 years	50 (63.3)	29 (36.7)	0.053	1.670	0.992–2.810
>20 years	3 (42.9)	4 (57.1)	0.680	0.726	0.159–3.312
***HbA1c***					
< 7%	8 (53.3)	7 (46.7)	0.997	0.998	0.352–2.827
≥ 7%	150 (53.4)	131 (46.6)			
***Onset of ulcer***					
Spontaneous (reference)	124 (56.1)	97 (43.9)	0.197	1.347	0.857–2.116
Traumatic	56 (48.7)	59 (51.3)			
***Ulcer duration***					
≥ 1 month	138 (58.2)	99 (41.8)	**0.009**	1.892	1.177–3.042
< 1 month	42 (42.4)	57 (57.6)			
***Neuropathy***					
Yes	144 (55.0)	118 (45.0)	0.337	1.288	0.768–2.160
No	36 (48.6)	38 (51.4)			
***Peripheral artery disease***					
Yes	110 (62.5)	66 (37.5)	**0.001**	2.143	1.385–3.316
No	70 (43.8)	90 (56.3)			
***Presence of Gangrene***					
Yes	113 (63.5)	65 (36.5)	**< 0.001**	2.361	1.522–3.662
No	67 (42.4)	91 (57.6)			
***Wound infection***					
Yes	142 (55.0)	116 (45.0)	0.327	1.289	0.776–2.140
No	38 (48.7)	40 (51.3)			

OR = Crude Odds Ratio (univariate logistic regression), p = probability of committing type 1 error (univariate logistic regression), C.I. = Confidence Interval.

**Table 3:** Relationship between comorbid complications and anemia.

	Anemia		P value	OR	95% C.I for OR
	Yes n (%)	No n (%)			
***Proteinuria***					
Yes	78 (64.5)	43 (35.5)	**0.003**	2.041	1.285–3.242
No	96 (47.1)	108 (52.9)			
***Blood culture***					
Positive	48 (59.3)	33 (40.7)	0.131	1.502	0.886–2.549
Negative	91 (49.2)	94 (50.8)			
***Osteomyelitis***					
Yes	59 (64.8)	32 (35.2)	**0.006**	2.018	1.218–3.342
No	106 (47.7)	116 (52.3)			
***Vascular Stenosis***					
None	52 (41.9)	72 (58.1)			
Mild	39 (50.0)	39 (50.00	0.263	1.385	0.783–2.447
Moderate	52 (64.2)	29 (35.8)	**0.002**	2.483	1.393–4.423
Severe	27 (75.0)	9 (25.0)	**0.001**	4.154	1.803–9.569
***Hypertension***					
Yes	101 (52.9)	90 (47.1)	0.770	0.938	0.608–1.446
No	79 (54.5)	66 (45.5)			
***Shock at presentation***					
Yes	25 (62.5)	15 (37.5)	0.230	1.516	0.768–2.991
No	155 (52.4)	141 (47.6)			
***Hyperglycemic emergency***					
Yes	67 (54.5)	56 (45.5)	0.802	1.059	0.678–1.653
No	113 (53.1)	100 (46.9)			
***Hypoglycemia***					
Yes	25 (62.5)	15 (37.5)	0.230	1.516	0.768–2.991
No	155 (52.4)	141 (47.6)			
***Stroke***					
Yes	24 (75.0)	8 (25.0)	**0.014**	2.846	1.240–6.535
No	156 (51.3)	148 (48.7)			
***Heart failure***					
Yes	17 (73.9)	6 (26.1)	0.050	2.607	1.002–6.788
No	163 (52.1)	150 (47.9)			
***Renal impairment***					
Yes	47 (71.2)	19 (28.8)	**0.002**	2.567	1.432–4.604
No	132 (49.1)	137 (50.9)			

OR = Crude Odds Ratio (univariate logistic regression), p = probability of committing type 1 error (univariate logistic regression), C.I. = Confidence Interval.

**Table 4:** Relationship between anemia and diabetic foot outcomes.

	Anemia		P value	OR	95% C.I for OR
	Yes n (%)	No n (%)			
***Satisfactory healing***					
Yes	40 (22.2)	55 (35.3)	**0.009**	0.525	0.324–0.849
No	140 (77.8)	101 (64.7)			
***Endpoint***					
Died	44 (27.8)	25 (17.5)	**0.034**	1.822	1.047–3.171
Survived	114 (72.2)	118 (82.5)			
***Amputation***					
Yes	66 (41.8)	43 (30.1)	**0.036**	1.668	1.035–2.689
No	8 (22.2)	28 (69.9)			

OR = Crude Odds Ratio (univariate logistic regression), p = probability of committing type 1 error (univariate logistic regression), C.I. = Confidence Interval.

**Table 5:** Results of multivariate regression analysis to determine independent predictors of anemia in the population.

Variable	P value	OR	95% C.I. for OR	
			Lower	Upper
Ulcer duration ≥ 1 month	0.504	1.217	0.684	2.164
Presence of PAD	0.584	1.219	0.600	2.473
Foot gangrene	0.100	1.575	0.916	2.709
Proteinuria	0.249	1.400	0.790	2.479
Osteomyelitis	0.380	1.311	0.716	2.400
Stroke	0.063	2.472	0.953	6.409
Renal impairment	0.239	1.548	0.748	3.207
Moderate arterial stenosis	0.352	0.586	0.190	1.806
Severe arterial stenosis	0.541	0.723	0.256	2.042

PAD = peripheral artery disease, OR = adjusted odds ratio (multivariate logistic regression), p = probability of committing type 1 error (multivariate logistic regression), C.I. = Confidence interval.

## Discussion

The present study showed that anemia is common in Nigerians hospitalized for DFU and is associated with adverse outcome including poor wound healing, amputation and increased mortality rate. According to earlier studies, anemia is common, albeit often unrecognized, in patients with DM independent of renal impairment [13–16]. The 53.6% prevalence of anemia found in this study, is lower than the 61.8% reported by Ekpebegh et al [17] in a study of forty-two hospitalized Nigerians with DFU. However, in that study anemia was defined as a hemoglobin (Hb) concentration of less than 10g/dL compared with less than 12g/dL used in the index study. This may account for the higher prevalence of anemia reported. Using Receiver Operating Curve (ROC) analysis, an Hb cut-off of 12.3g/dl (female) and 12.1g/dl (male) in one study, was reported to better identify patients with DFU who are at high risk of adverse outcomes [12].

Nearly half of our patients with anemia required blood transfusion. The reason for the high rate of blood transfusion is likely to be multifactorial. Although the mechanism for the development of anemia in patients with DFU is yet to be fully established, some authors have found a relationship between clinical grade of ulcer and anemia among their subjects [16, 18]. Our cohort with DFU included all Wagner grades (1–5), with grade 4 being the most prevalent. Ulcer grade ≥4 was associated with the highest mortality in the study by Ekpebegh et al [17].

Chronic inflammation, diabetic nephropathy and malnutrition are other factors suggested to cause anemia in patients with DFU. Of these additional factors, chronic inflammation is most frequently associated with anemia in patients with diabetes, especially in those with DFU [19]. One explanation for chronic inflammation induced anemia is the ability of inflammatory cells, especially macrophages, to mop up available iron and store it in ferritin, thereby preventing the transport of iron to the bone marrow [20].

We did not measure serum levels of inflammatory markers in this study. Nonetheless, the fact that three-quarter of our patients had infected DFU and more than half of those with anemia had positive blood cultures, consistent with septicemia, we could speculate that increased concentration of the inflammatory markers contributed to anemia in our patients. Khanbai et al [21] reported that inflammatory and infective processes leading to progression of DFU were associated with decreased hemoglobin and increased C-reactive protein (CRP) levels.

Our study revealed a significant association between anemia and renal impairment in our cohort. Patients with anemia in this study were 2.5 times more likely to have renal impairment compared with their counterparts without anemia. More than half of the patients with anemia also had proteinuria. We concluded therefore, that diabetic nephropathy (DN) was a significant determinant of anemia in the index study. This concurs with the findings of Chuan et al [12] who also reported a significant association between anemia and DN among their patients with DFU. Reports from previous studies indicate that anemia complicating chronic kidney disease (CKD) due to diabetes, tended to be more severe and occur much earlier than in nondiabetic CKD [22,23]. Furthermore, CKD is an independent risk factor for DFU [24].

Apart from renal impairment, malnutrition may have contributed to the development of anemia in our patients. In fact, both CKD and DFU are chronic debilitating illnesses that are associated with poor dietary intake leading to nutritional deficiencies. The latter manifest with a multitude of systemic symptoms including anemia [25]. Although not explored in this study, blood loss from hookworm infestation is also a recognized cause of anemia in our environment especially in those who walk barefooted [18].

We found a significant relationship between anemia, peripheral arterial disease (PAD) and stroke in this study. There are few studies on the effect of anemia on PAD in patients with DFU. In the study by Chuan et al [12], there was no relationship between anemia and PAD. The authors however, found a significant association between anemia and stroke among their patients, which is consistent with our finding. In patients with diabetes, anemia confers increased risk of stroke, hypertension, ischemic heart disease and nephropathy [10].

Anemia in the present study was associated with poor wound healing, amputation and increased mortality. Satisfactory wound healing requires adequate blood flow to provide oxygen and other essential nutrients to damaged tissues. Cahn et al [26] reported increased red cell deformability in patients with DFU, which may lead to decreased blood flow and delayed wound healing. The decreased oxygenation caused by anemia further compounds the problem.

The finding of high rates of amputation and mortality in patients with anemia in this study is in contrast with the report by Wright et al [9], who did not find any significant association between anemia, and either minor or major amputation or mortality. However, Wright and colleagues attributed their findings to the small number of patients included in their study and the existence of a proactive multidisciplinary approach to diabetic foot care at their center. In the presence of anemia, limb ischemia caused by PAD is often aggravated because of poor oxygen delivery to the periphery. In addition, studies indicate that the hyperkinetic state accompanying anemia may heighten the expression of the endothelial adhesion molecule gene leading to thrombus formation further impairing peripheral circulation [27]. All of these could ultimately lead to gangrene of the limb culminating in amputation. Our findings clearly demonstrate a significant association between anemia and adverse outcome. However, this does not infer a causal relationship between anemia and poor outcome. There are few Nigerian studies designed to investigate the impact of anemia on outcome in patients with DFU. In the study by Ekpebegh et al [17], mortality among their patients with DFU was significantly associated with anemia and leukocytosis. A more recent study from Brazil also reported significant association between anemia and amputation, and mortality among patients with DFU [28], in keeping with our findings.

The strength of our study is that, to the best of our knowledge, it is the first multicenter study to assess the impact of anemia on disease outcome in Nigerians with DFU. However, the study also has limitations. These include our inability to conduct detailed assessment of our patients with anemia to establish the specific type of anemia and failure to measure inflammatory markers in patients with infected DFU, which would have shed more light on the relationship between anemia and chronic inflammation in this study. Despite these limitations, the result of this study provides baseline data on the prevalence of anemia and its impact on disease outcome in Nigerians with DFU for future studies to build on.

## Conclusion

In this study, we demonstrated that anemia is common among Nigerians hospitalized for DFU. The most prevalent comorbidity associated with anemia was renal impairment. We found a significant association between anemia and poor wound healing, amputation and mortality. Future studies should explore whether prompt correction of anemia in subjects hospitalized for DFU would improve outcome.

## Supporting information

S1 Data Dataset of the subjects supporting the findings of this article on excel spreadsheet. (XLSX)
